# Delineating a New Heterothallic Species of *Volvox* (Volvocaceae, Chlorophyceae) Using New Strains of “*Volvox africanus*”

**DOI:** 10.1371/journal.pone.0142632

**Published:** 2015-11-12

**Authors:** Hisayoshi Nozaki, Ryo Matsuzaki, Kayoko Yamamoto, Masanobu Kawachi, Fumio Takahashi

**Affiliations:** 1 Department of Biological Sciences, Graduate school of Science, University of Tokyo, Tokyo, Japan; 2 Center for Environmental Biology and Ecosystem Studies, National Institute for Environmental Studies, Tsukuba, Ibaraki, Japan; 3 Ritsumeikan University, College of Life Sciences, Kusatsu, Shiga, Japan; Donald Danforth Plant Science Center, UNITED STATES

## Abstract

The volvocine algae represent an excellent model lineage in which to study evolution of female and male genders based on comparative analyses of related species. Among these species, *Volvox carteri* has been extensively studied as a model of an oogamous and complex organism. However, it may have unique derived features that are not present in other species of *Volvox*. Therefore, information regarding the characteristics of sexual reproduction of other species of *Volvox* is also important. In 1971, Starr studied four types of sexuality in several global strains identified as *Volvox africanus*; however, further taxonomic studies of these strains have been lacking, and strains of three of the four sexual types are not available. Here, we studied the morphology, sexual reproduction, and taxonomy of two *V*. *africanus*-like species isolated recently from Lake Biwa, Japan. These two species were very similar to two sexual types described by Starr in 1971: one producing dioecious sexual spheroids in heterothallic strains and the other forming both male spheroids and monoecious spheroids in a single strain. The former species produced zygotes with a reticulate cell wall, whereas a smooth zygote wall was observed in the latter species as in *V*. *africanus* previously reported from various localities around the world. Our multigene phylogenetic analysis demonstrated that these are sister species to each other. However, the presence of a compensatory base change in the most conserved region of the secondary structure of nuclear ribosomal DNA internal transcribed spacer-2, hybrid inviability demonstrated by intercrossing experiments, and morphological differences in the density of abutment between the gelatinous material of adjacent cells (individual sheaths) in the spheroid supported the recognition of the two species, *V*. *africanus* having a smooth zygote wall and *V*. *reticuliferus* Nozaki sp. nov. having a reticulate zygote wall.

## Introduction

*Volvox* is a fantastic green alga that was taxonomically described as a genus by Linnaeus [1]. Smith [2] recognized approximately 20 species that were subdivided into four sections based on morphological differences in cells and gelatinous matrix structures of spheroids. *Volvox* sect. *Merrillosphaera* is distinguished from other sections of the genus *Volvox* by a lack of cytoplasmic bridges between adult cells in spheroids, and includes about eight species [2–5]. Recent chloroplast multigene phylogenies have demonstrated that the genus *Volvox* is polyphyletic, representing four separate lineages, two of which include species of *Volvox* sect. *Merrillosphaera* sensu Smith [6–8].

The volvocine algae that include the unicellar *Chlamydomonas reinhardtii* Dangeard and milticelluar *Volvox* represent an excellent model lineage in which to study evolution of female and male genders based on comparative analyses of related species [9–12]. Among such algae, *Volvox* (sect. *Merrillosphaera*) *carteri* Stein has been studied extensively as a model of the most advanced or oogamous and complex organism [10, 13]. However, as the most advanced member of the volvocine algae, *V*. *carteri* may have unique derived features that are not present in other *Volvox* species. Therefore, information regarding other *Volvox* species, in particular those related to *V*. *carteri*, is needed to understand the main evolutionary tendency in the volvocine lineage.

Starr [14] reported four types of sexual reproduction in several strains identified as “*Volvox* (sect. *Merrillosphaera*) *africanus* G. S. West” originating from Australia, South Africa, the USA, and India. However, further taxonomic studies of these strains have not been carried out. Unfortunately, only strains belonging to one of the four sexual types are available (dioecious, heterothallic type of Starr [14]): Darra 4 (UTEX 1890; http://www.utex.org/default.asp) and Darra 6 (NIES-863 = UTEX 1891 [15], http://mcc.nies.go.jp/localeAction.do?lang=en), but the internal transcribed spacer-2 (ITS-2) of nuclear ribosomal DNA (rDNA) sequences were available for phylogenetic analyses [16]. As these strains are > 50 years old, reexamination of the characteristics of sexual reproduction is difficult or impossible [17]. Therefore, *V*. *africanus*-like stains newly established from field-collected samples are needed to study the taxonomic and evolutionary significance of the diversity of sexual types in “*V*. *africanus*” [14].

Recently, two *V*. *africanus*-like species were isolated from Lake Biwa, Japan. These were very similar to two of the four sexual forms [14]: one produced both male spheroids and monoecious spheroids developing in a single strain (homothallism), whereas the other produced only male or female spheroids in a single strain (heterothallism). Our molecular and morphological analyses identified the former as *V*. *africanus* and the latter as a new species, *Volvox reticuliferus* Nozaki sp. nov. This report describes the morphology, sexual reproduction, molecular phylogeny, and taxonomy of these two species.

## Materials and Methods

Water samples (24.5°C, pH 8.4) from which *V*. *africanus* strain 2013-0703-VO4 and *V*. *reticuliferus* strains 2013-0703-VO1, VO2 and VO3 were isolated were collected from the shore (35°04’26”N, 135°55’55”E) of Lake Biwa, Shiga Prefecture, Japan, on 3 July 2013. Collection of the samples from Lake Biwa was permitted by Biodiversity Strategy Promotion Office, Department of Environment of Lake Biwa, Shiga Prefecture, Japan. Clonal culture strains were established by the pipette-washing method [18], and grown in AF-6 medium [15, 19] or AF-6/3 medium [20]. The *Volvox* cultures were grown in screw-capped tubes (18 × 150 mm) containing about 11 mL medium at about 20°C or 25°C on a 14-h light:10-h dark schedule under cool-white fluorescent lamps at an intensity of 80–100 μmol∙m^–2^∙s^–1^. In addition, F_1_ progeny strains were obtained as previously described [3, 13] from the two species: strain VO4-F1-1 from *V*. *africanus* strain 2013-0703-VO4, and strains VO123-F1-6 (female) and F1-7 (male) from *V*. *reticuliferus* strains 2013-0703-VO1, VO2 and VO3. These three F_1_ strains were also examined as described above. The new wild strains of *V*. *africanus* and *V*. *reticuliferus* and their F_1_ progeny strains (S1 Table) are available from Microbial Culture Collection at the Institute for National Environmental Studies [15] (http://mcc.nies.go.jp/ webcite) as NIES-3780~3786. For comparison, we used “*V*. *africanus*” strains UTEX 1890 and 1891 (Darra 4 and Darra 6 [14], respectively [21]) that we [12] had already obtained from the Culture Collection of Algae at the University of Texas at Austin (UTEX, USA [21], http://www.utex.org/). In addition, we also used “*V*. *africanus*” strain UTEX 2907 (no information at UTEX) that had also been obtained previously (Oct. 2011) from UTEX. These three strains were grown in AF-6 medium as described above.

For observation of asexual spheroids, aliquots of actively growing culture (about 0.5 mL) were inoculated into fresh AF-6 medium or AF-6/3 medium every 10–20 days. A BX60 microscope (Olympus, Tokyo, Japan) equipped with Nomarski interference optics was used for light microscopic examinations. Transmission electron microscopy (TEM) of asexual spheroids was carried out as previously described [22] except using a JEM-1010 electron microscope (JEOL, Tokyo, Japan).

Sexual spheroids developed spontaneously in each strain of the two species when the culture was repeatedly inoculated (every 5–7 days). To enhance sexual induction, the cultures were grown in USVT medium [20] diluted with double-distilled water (USVT/3 medium) at 25°C on a 14-h light:10-h dark schedule under cool-white fluorescent lamps at an intensity of 200–220 μmol∙m^–2^∙s^–1^. Sexually induced male and female cultures were mixed for formation of zygotes in heterothallic strains. Upon the mixture of sexually induced male and female cultures (in the heterothallic species *V*. *reticuliferus*) or just after the formation of sexual spheroids (in the homothallic species *V*. *africanus*), a half to equal volume of new USVT/3 medium was added to the culture for zygote maturation.

For intercrossing experiments, 17–28 male spheroids of *V*. *africanus* strain VO4-F1-1 induced in USVT/3 medium were isolated with a micropipette and washed twice with new AF-6/3 medium, and only male spheroids were inoculated into the induced female cultures of *V*. *reticuliferus* strain VO123-F1-6 in 7–11 mL USVT/3 medium. Then, a half to equal volume of new USVT/3 medium was added to the culture for zygote maturation.

Extracting total DNA and sequencing the five chloroplast genes (*rbcL*, *atpB*, *psaA*, *psaB*, and *psbC* genes) and ITS-2 regions of nuclear rDNA (nuclear rDNA ITS-2) were performed as described previously [23–25]. For chloroplast multigene phylogeny, 6021 base pairs of the concatenated exon sequences of the five chloroplast genes from *V*. *africanus* strain 2013-0703-VO4 (LC090149–53), *V*. *reticuliferus* strain 2013-0703-VO2 (LC090154–58), plus 36 operational taxonomic units (OTUs) [8] belonging to the *Eudorina* group (*Eudorina*, *Pleodorina*, and *Volvox* excluding section *Volvox*), *Yamagishiella*, and VPC clade (*Volvox* sect. *Volvox*, *Platydorina*, and *Colemanosphaera*) were unambiguously aligned by ClustalX [26]. The alignment (available from TreeBASE: http://www.treebase.org/treebase-web/home.html; study ID: S18363) was subjected to Bayesian inference (BI) with MrBayes 3.2.5 [27] using evolutionary models unlinked and selected using MrModeltest 2.3 [28] for each codon position in the five concatenated genes. In addition, bootstrap analyses [29] were performed with 1000 replicates by the maximum-likelihood (ML) method with RAxML ver. 7.4.2 [30] using evolutionary models unlinked and selected for each codon position in the five concatenated genes as in BI and by the maximum-parsimony (MP) method using a heuristic search with the stepwise addition of 10 random replicates (with the tree bisection-reconnection branch-swapping algorithm) using PAUP 4.0b10 [31]. *Yamagishiella* and the VPC clade were treated as the outgroup because they are positioned basally to the *Eudorina* group [8, 23].

Alignment of nucleotide sequences for nuclear rDNA ITS-2 from *Volvox reticuliferus*, *V*. *africanus*, *V*. *africanus*-like strains studied [14, 16], “*V*. *africanus*” strain UTEX 2907, four related species of *Volvox* (*V*. *ovalis*, *V*. *tertius*, *V*. *spermatosphaera* and *V*. *dissipatrix* (Shaw) Printz [5]) (S1 Table) was performed based on the secondary structures and using ClustalX. The secondary structures of ITS-2 were predicted using CentroidFold [32, 33] and RNAfold at the RNAfold WebServer ([34], http://rna.tbi.univie.ac.at/cgi-bin/RNAfold.cgi), and the secondary structure models [35, 36]. Identical sequences were treated as a single OTU, and an alignment of 646 base pairs from 11 OTUs (14 strains, S1 Table) was obtained (available from TreeBASE; study ID: 18363). The alignment was subjected to phylogenetic analyses by ML (with the T92+G model selected) and MP (with branch-and-bound search) as described previously [20]. *V*. *ovalis*, *V*. *tertius*, *V*. *spermatosphaera*, and *V*. *dissipatrix* were treated as the outgroup because these four species form a monophyletic group that is sister to *V*. *africanus* [5, 8].

ITS-2 secondary structures were compared to detect compensatory base changes (CBCs) of nuclear rDNA ITS-2.

### Nomenclature

The electronic version of this article in Portable Document Format (PDF) in a work with an ISSN or ISBN will represent a published work according to the International Code of Nomenclature for algae, fungi, and plants; hence, the new names contained in the electronic publication of a PLOS ONE article are effectively published under that Code from the electronic edition alone, so there is no longer any need to provide printed copies.

## Results

### Asexual spheroids

**Fig 1:**
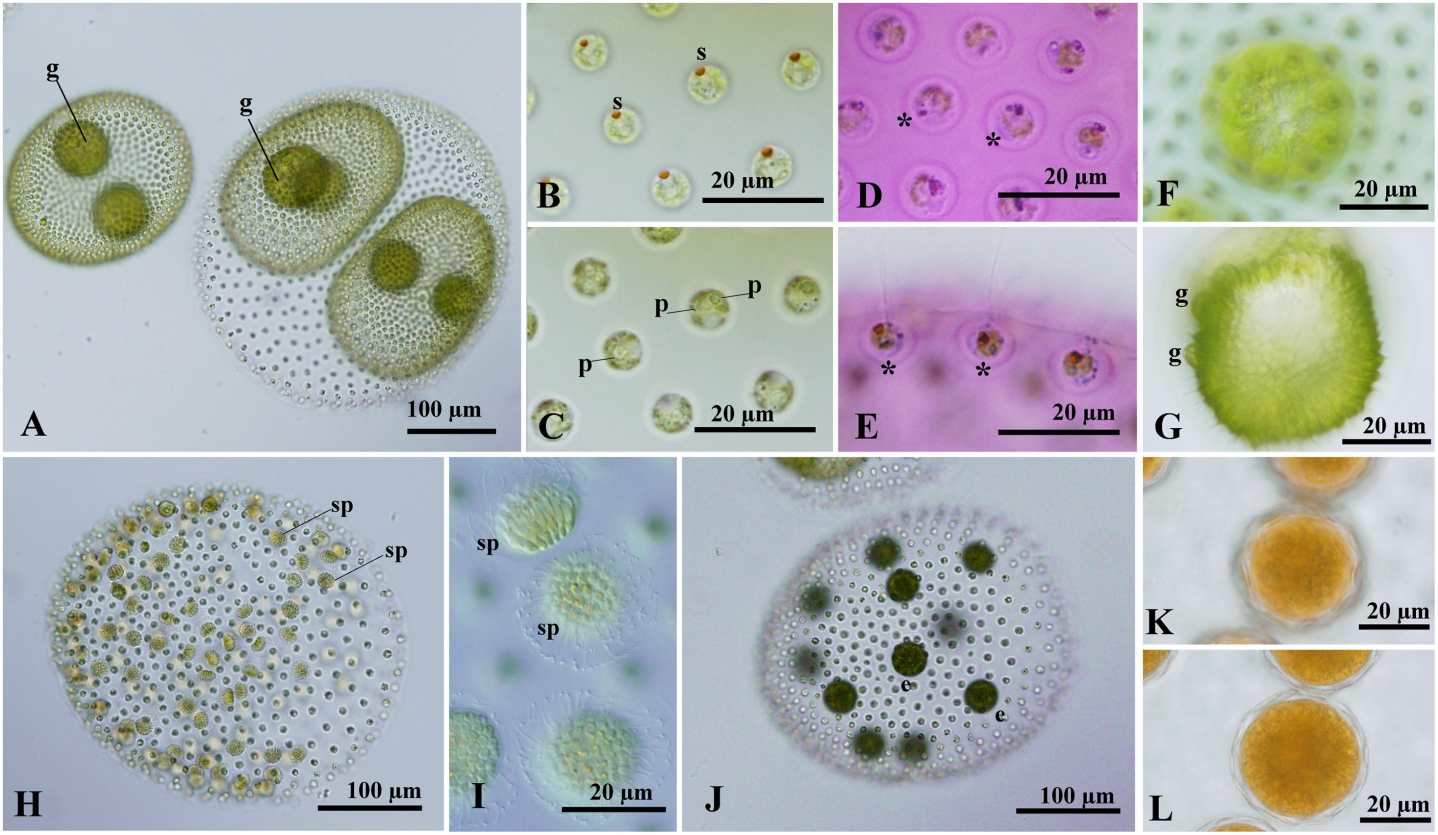
Nomarski interference microscopy of *Volvox reticuliferus* Nozaki sp. nov. (A) Surface view of asexual spheroids showing small somatic cells and larger reproductive cells (gonidia). (B, C) Somatic cells in the anterior portion of asexual spheroid showing lack of cytoplasmic bridges between cells. (B) Surface view showing stigma. (C) Optical section showing pyrenoid. (D, E) Asexual spheroid stained with dilute aniline blue, showing a broad secondary boundary layer (asterisk) of the gelatinous matrix surrounding each somatic cell. (D) Front view of somatic cells. (E) Side view of somatic cells. (F) Surface view of chloroplast of gonidium of asexual spheroid. Note presence of radial striations in the chloroplast surface. (G) Pre-inversion stage. Note morphological differentiation of gonidia of the next generation. (H, I) Mature male spheroids with sperm packets. (J) Female spheroid with eggs. (K, L) Two views of mature zygotes with a reticulate wall. Abbreviations: e, egg; g, gonidium; p, pyrenoid; s, stigma; sp, sperm packet. (A-C, H, I) Strain VO123-F1-7. (D-G) Strain VO123-F1-6. (D-G) Strain 2013-0703-VO2. (K, L) Strains 2013-0703-VO2x3.

*Volvox africanus* strains 2013-0703-VO4 and VO4-F1-1, and *V*. *reticuliferus* strains 2013-0703-VO1, VO2 and VO3, and VO123-F1-6 and F1-7 can be assigned to *V*. *africanus* based on the morphology of the asexual spheroids, as delineated previously [2, 5] except for individual sheaths. The asexual spheroids of both species were ovoid or ellipsoidal with a broad anterior face, and contained 800–3000 cells arranged in a single layer at the periphery of the gelatinous matrix, measuring up to 350–400 μm in length (Fig 1A; S1A Fig). The somatic cells were embedded in individual sheaths of the gelatinous matrix, and nearly spherical in shape, lacking cytoplasmic bridges between them (Fig 1B–1E; S1B–S1D Fig). The cells had two equal flagella, two contractile vacuoles near the base of the flagella, and a cup-shaped chloroplast with a single stigma and a basal pyrenoid, measuring up to 7–8 μm in diameter. There was gradual diminution in stigma size from the anterior to posterior pole of the spheroids. In *V*. *reticuliferus*, the number of pyrenoids in somatic cells often increased to two or more (Fig 1C), whereas *V*. *africanus* almost always had a single pyrenoid (S1B Fig). Both species generally had two to four gonidia, or sometimes more, in an asexual spheroid (Fig 1A; S1A Fig). Two to four of the gonidia were distributed near the middle of the spheroid, and the remainder, when present, were located in the posterior half. The mature gonidia, measuring up to 60–65 μm in diameter, were spherical in shape and vacuolated, and had numerous contractile vacuoles and a large chloroplast occupying the whole cytoplasm and containing a dozen or more pyrenoids distributed randomly. The surface of the chloroplast showed numerous longitudinal fine striations that were radially arranged in top view (Fig 1F). Juvenile spheroids developed in pairs within the parent in both species (Fig 1A; S1A Fig).

Individual sheaths, the outermost layer of matrix that abuts that of the adjacent cell, of asexual spheroids of *V*. *africanus* were distinct and adhered to one another in such a way as to appear pentagonal, hexagonal, or heptagonal in front view due to mutual compression (S1C Fig). Within each individual sheath of a fully mature spheroid, the somatic cell was enclosed by a broad secondary boundary layer of matrix that appeared almost circular in front view (S1D Fig). In contrast, individual sheaths of asexual spheroids in *V*. *reticuliferus* strains 2013-0703-VO1, VO2 and VO3, and VO123-F1-6 and -7, as well as strains UTEX 1890, UTEX 1891, and UTEX 2709 of “*V*. *africanus*,” were almost confluent or indistinct even when stained with methylene blue or aniline blue (Fig 1D and 1E; S2 Fig); each somatic cell was enclosed by a broad secondary boundary layer of matrix that appeared almost circular in front view.

Differences in gelatinous matrix structures seen under the light microscope were also examined by TEM (S3 Fig). In *V*. *reticuliferus*, a dense secondary boundary layer of extracellular matrix encompassing each somatic cell was distinct and separated from that of the adjoining somatic cells (S3A–S3C Fig). This layer was apparently separated from the cellular envelope that tightly enclosed each somatic cell [37]. However, *V*. *africanus* had no such distinct dense layer (S3D–S3F Fig).

Individual sheaths appear to stain with methylene blue or aniline blue (Fig 1D and 1E; S1C and S1D Fig), but were not visibly different under TEM. In *V*. *africanus*, the extracellular matrix formed an angular space for each somatic cell (S3D–S3F Fig), possibly corresponding to individual sheaths on light microscopy (S1C and S1D Fig). On the other hand, individual sheaths of *V*. *reticuliferus* were not evident under TEM (S3A–S3C Fig).

In asexual reproduction of both species of *Volvox*, gonidia divided successively to develop a hollow plakea that became a juvenile spheroid after inversion. During the plakeal and inversion stages, the gonidia or gonidial initials of the next generation were evident (Fig 1G; S1E Fig).

### Sexual spheroids

Strains of *V*. *reticuliferus* were heterothallic, that is genetically either male or female. Male spheroids produced by strains 2013-0703-VO3 and VO123-F1-7 were ellipsoidal or cylindrical, containing 1000–1500 biflagellate somatic cells and 80–120 androgonidia that divided into plate-shaped sperm packets (Fig 1H and 1I). Female spheroids produced by strains 2013-0703-VO2 and VO123-F1-6 were ovoid with a broad anterior face, composed of 1000–2000 biflagellate somatic cells and 8–20 eggs (Fig 1J). After possible fertilization, the zygote developed a reticulate cell wall and turned reddish brown in color (Fig 1K and 1L). The mature zygote measured 33–39 μm in diameter.

Two types of sexual spheroid, male and monoecious spheroids, were produced within a culture or even in a single parental spheroid of the present *V*. *africanus* strains (2013-0703-VO4 and VO4-F1-1) (S1F Fig). Male spheroids were ellipsoidal to cylindrical, containing 1000–3200 biflagellate somatic cells and 100–260 androgonidia that divided into plate-shaped sperm packets (S1G Fig). Monoecious spheroids were ovoid with a broad anterior face, composed of approximately 1500 biflagellate somatic cells, 15–47 eggs, and 1–4 plate-shaped sperm packets (S1H and S1I Fig). After possible fertilization, the zygote developed a smooth cell wall and turned reddish brown in color (S1J and S1K Fig). The mature zygote measured 31–36 μm in diameter.

### Intercrossing between *V*. *africanus* male spheroids and *V*. *reticuliferus* female spheroids

Male spheroids of the sexually induced *V*. *africanus* strain VO4-F1-1 were isolated by micropipette and mixed with sexually induced *V*. *reticuliferus* female spheroids (strain VO123-F1-6). After several days, a small portion (< 20%) of eggs in the female spheroids secreted a cell wall. Such walled eggs were spherical in shape and had an almost smooth cell wall (S4A and S4D Fig). Approximately 2 weeks after mixing of gametes, part of the walled eggs turned reddish brown in color and had a thick cell wall that was smooth or weakly reticulate (S4B and S4E Fig). However, no reddish brown eggs were observed 3 weeks after mixing; all of the walled eggs lost their reddish or greenish color and almost all disintegrated, with rupture of their walls (S4C and S4F Fig). As no eggs of *V*. *reticuliferus* female spheroids developed into a reddish brown cell with a thick cell wall without male spheroids (not shown), the thick-walled reddish brown eggs in the female spheroids were possible hybrid zygotes.

### Molecular phylogenetic analyses

**Fig 2:**
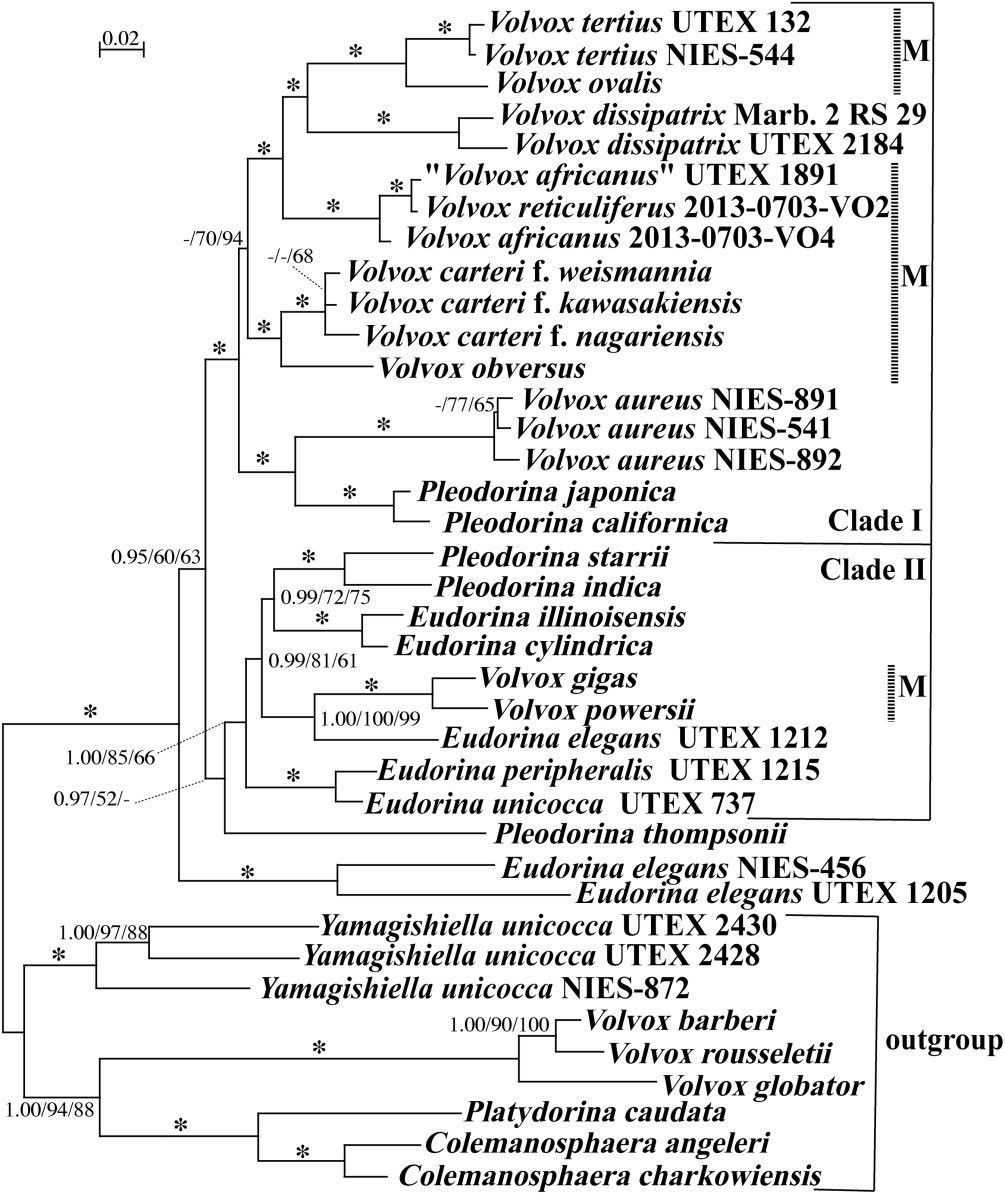
Phylogenetic positions of *Volvox reticuliferus* Nozaki sp. nov. and *V*. *africanus* G. S. West within the advanced members of the Volvocaceae (*Eudorina* group [8, 23]), as inferred from 6021 base pairs of five chloroplast genes. The tree was constructed by Bayesian inference unlinked GTR+I+G, F81+I+G and GTR+I+G models for the first, second and third codon positions in the five concatenated genes, respectively. Branch lengths are proportional to the genetic distances, which are indicated by the scale bar above the tree. Numbers on the left, middle, or right side at the branches represent posterior probabilities (PP) of BI (≥0.95), bootstrap values (≥50%, based on 1,000 replicates) obtained with the maximum likelihood and maximum parsimony analyses, respectively. Asterisks at the branches indicate 1.00 PP and 100% bootstrap values by the two methods. For details of the methods, see the text. Longitudinal dashed lines associated with “M” represent species of *Volvox* sect. *Merrillosphaera* sensu Smith [2].

**Fig 3:**
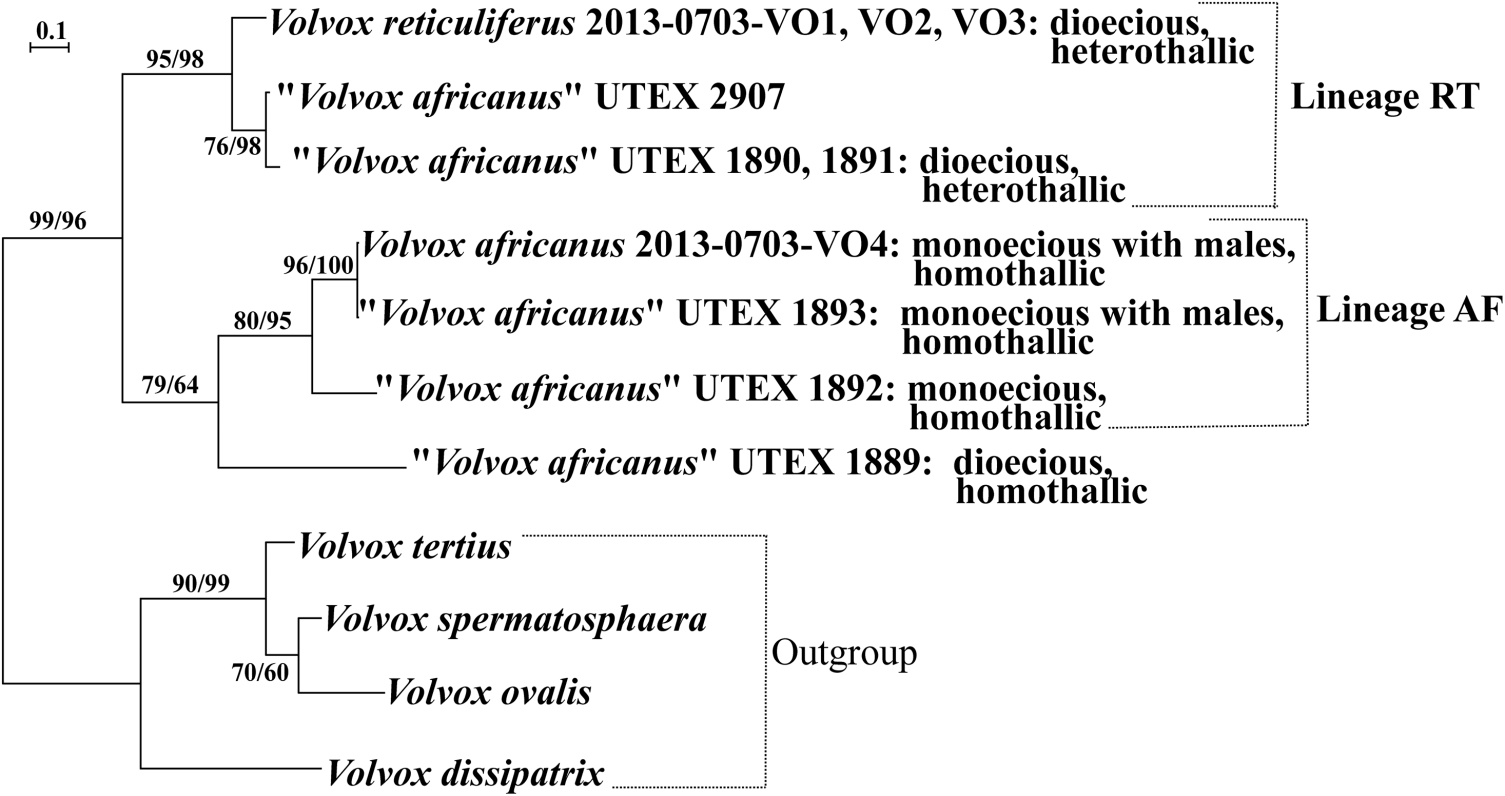
Phylogenetic relationships of various strains of *Volvox reticuliferus* Nozaki sp. nov. and *V*. *africanus* G. S. West, as inferred from internal transcribed spacer region 2 (ITS-2) of nuclear ribosomal DNA. The tree was constructed by the maximum likelihood (ML) method (based on T92+G model). Branch lengths are proportional to the genetic distances, which are indicated by the scale bar above the tree. Numbers on the left or right side at the branches represent bootstrap values (≥50%, based on 1,000 replicates) obtained with the ML calculation and maximum parsimonious analysis (using a branch-and-bound search), respectively. Sexual types (S1 Table) are shown right to the strain designations.

Based on the present phylogenetic analyses of chloroplast genes, species of *Volvox* sect. *Merrillosphaera* sensu Smith were positioned in two large clades (clades I and II) (Fig 2) as in previous studies [5, 24]. Clade I was supported by 1.00 Bayesian posterior probabilities (BPP) and 100% bootstrap values (BV) in maximum likelihood (ML) and most parsimonious (MP) analyses, and comprised six species of *Volvox* sect. *Merrillosphaera* sensu Smith (including *V*. *africanus* and *V*. *reticuliferus*), *V*. *aureus* Ehrenberg, *V*. *dissipatrix*, and two homothallic *Pleodorina* species (*P*. *californica* Shaw and *P*. *japonica* Nozaki [38]). Within clade I, *V*. *africanus* and *V*. *reticuliferus* (including “*V*. *africanus*” strain UTEX 1891; see the discussion section below) constituted a robust monophyletic group (with 1.00 BPP and 100% BVs in ML and MP analyses) to which a robust clade composed of *V*. *dissipatrix*, *V*. *ovalis*, and *V*. *tertius* was sister (Fig 3). Clade II was supported with moderate to weak support values (1.00 BPP and 66–85% BVs) and comprised two other species of *Volvox* sect. *Merrillosphaera* sensu Smith [*V*. *gigas* Pocock and *V*. *powersii* (Shaw) Printz], two heterothallic *Pleodorina* species [*P*. *starrii* Nozaki et al. and *P*. *indica* (Iyengar) Nozaki [24, 38]] and five *Eudorina* strains (Fig 2).

In phylogenetic analyses of the ITS-2 of nuclear rDNA sequences, the present new strains of *V*. *reticuliferus* and *V*. *africanus*, five strains (UTEX 1889, 1890, 1891, 1892, and 1893) representing the four sexual types described by Starr [14] and “*V*. *africanus*” strain UTEX 2907 (sexuality unknown) formed a robust monophyletic group (with 96–99% BVs in ML and MP analyses) (Fig 3). This group was subdivided into two sister clades as reported by Coleman [16] except for the new strains and “*V*. *africanus*” strain UTEX 2907: one (lineage RT) composed of *V*. *reticuliferus* strains 2014-0703-VO1, VO2 and VO3, “*V*. *africanus*” strain UTEX 2907, and two heterothallic and dioecious strains of “*V*. *africanus*” [UTEX 1890 and 1891 (Darra 4 and 6, respectively) [14]], and the other consisting of three homothallic as described by Starr [14] (“*V*. *africanus*” strains UTEX 1889, 1892, and 1893) and *V*. *africanus* strain 2013-0703-VO4 (Fig 3). Although the former clade was resolved with high BVs (95–98%), the latter was supported with moderate to weak BVs (64–79%). Within the latter clade, “*V*. *africanus*” strain UTEX 1889 (Mo-1-Ea, homothallic, dioecious type [14]) was sister to the monophyletic group (lineage AF, supported with 80–95% BVs) composed of the other strains.

### Compensatory base changes (CBCs) of nuclear rDNA ITS-2

**Fig 4:**
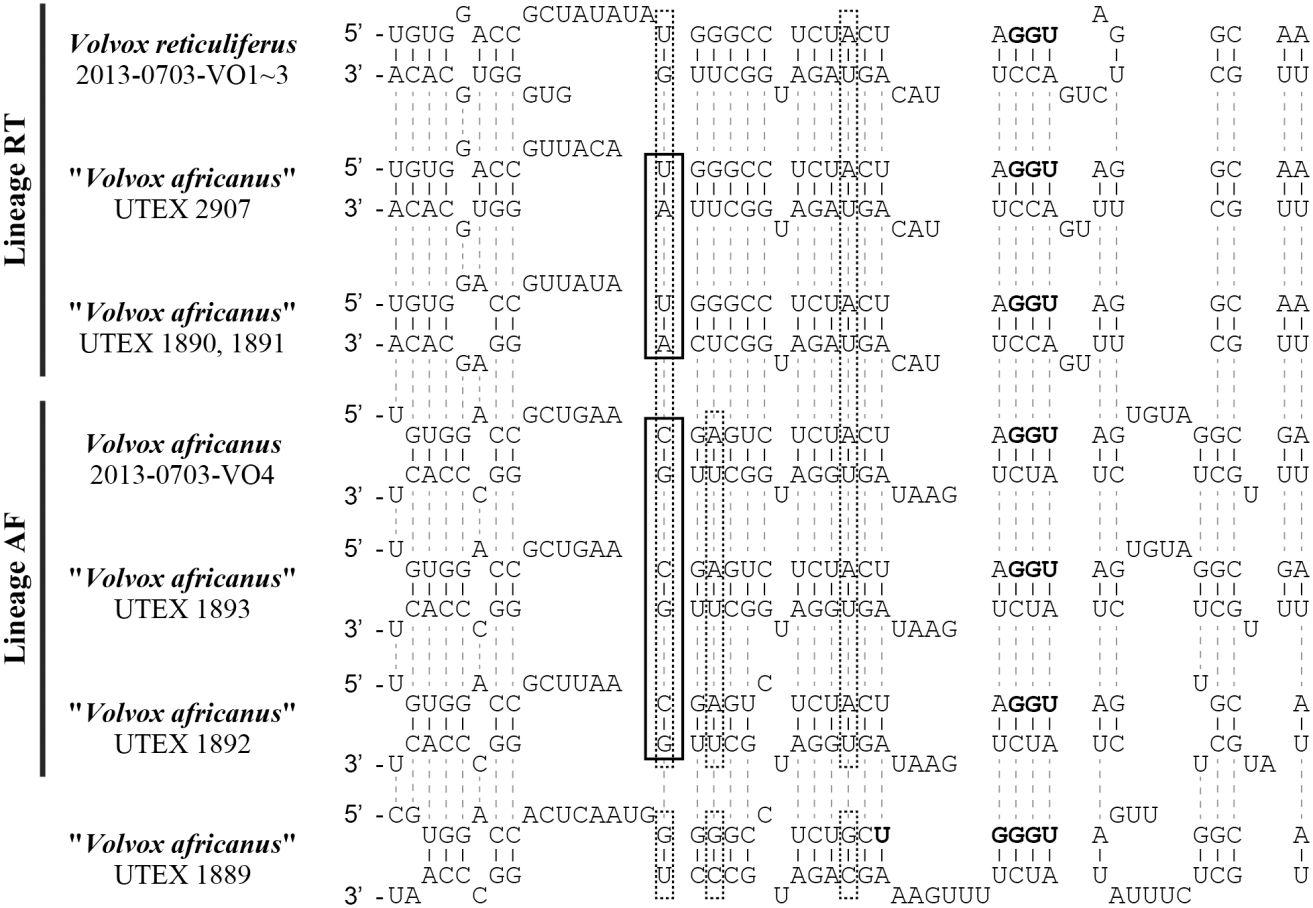
Comparison of the most conserved region (near the YGGY motif of helix III [39]) of nuclear rDNA ITS-2 secondary structure between lineage RT (corresponding to *Volvox reticuliferus* Nozaki sp. nov., see Discussion of the main text), lineage AF (including *V*. *africanus* G. S. West) and “*V*. *africanus*” strain UTEX 1889 (Fig 3). Note the modified YGGY motif (boldface). For secondary structures of ITS-2, see S5 Fig. Compensatory base changes between lineages RT/AF and “*V*. *africanus*” strain UTEX 1889 are marked with dotted open boxes, and those between lineages RT and AF are indicated by open boxes.

Between OTUs within the lineage RT or AF (Fig 3), CBCs were not detected in the ITS-2 sequence encompassing the YGGY motif (boldface in S5 Fig) in helix III, which is the most highly conserved region of nuclear rDNA ITS-2 [35, 39]. The nuclear rDNA ITS-2 sequence of “*V*. *africanus*” strain UTEX 1889 in this region showed two or three CBCs compared to that of the lineages RT or AF, respectively (Fig 4). However, a single CBC was present in the nuclear rDNA ITS-2 sequence between lineages RT and AF except for ITS-2 sequence comparison between a single OTU (*V*. *reticuliferu*s strains 2013-0703-VO1, VO2 and VO3) of linage RT and lineage AF (Fig 4).

## Discussion

*Volvox africanus* was originally described by West [40] based on material collected in Lake Albert Nyanza, a shallow lake on the border between Uganda and Congo in the Great Rift Valley, Africa (S2 Table). West [40] observed asexual spheroids of *V*. *africanus* and a single male spheroid of an uncertain species of *Volvox*. He later examined possible female spheroids and zygotes with a smooth wall of *V*. *africanus* originating from small ponds in Ussangu Desert in the African region formerly known as “German East Africa” [41]. Rich and Pocock [42] reported asexual spheroids of *V*. *africanus* collected in South Africa. Shaw [43] and Starr [14] demonstrated that both male and monoecious spheroids were produced in the same parental spheroid of *V*. *africanus* originating from the Philippines and India, respectively. Iyengar [44] observed both dioecious (male and female) and monoecious sexual spheroids of *V*. *africanus* originating from India. Although Starr [14] did not report zygotes of his strains of *V*. *africanus*, West [41], Shaw [43], and Iyengar [44] observed smooth zygote walls of *V*. *africanus* as in our *V*. *africanus* material from Japan (S1J and S1K Fig). In contrast, our heterothallic strains of *V*. *reticuliferus* formed mature zygotes with a reticulate wall (Fig 1K and 1L).

The asexual spheroids of our new strains of *V*. *africanus* and *V*. *reticuliferus* are consistent with those of *V*. *africanus* characterized by Smith [2] and Nozaki and Coleman [5] with regard to their shape, the number and development of gonidia or juvenile spheroids in them, and the early development of gonidia of the next generation during embryogenesis. However, the structure of the gelatinous matrix in *V*. *reticuliferus* is different from that of our Japanese strains of *V*. *africanus*. In our new strains of *V*. *africanus*, individual sheaths are distinct and appear pentagonal, hexagonal, or heptagonal in surface view due to mutual compression (S1C and S1D Fig). Rich and Pocock [42] observed distinct pentagonal or hexagonal individual sheaths in their South African *V*. *africanus* material. According to Iyengar [44], distinct pentagonal or hexagonal individual sheaths were also observed in the Indian material of *V*. *africanus* as well as the slide material of *V*. *africanus* studied by Shaw [43]. However, individual sheaths of our new strains of *V*. *reticuliferus* were confluent or indistinct even when stained with aniline blue (Fig 1D and 1E). This morphological difference was also confirmed by our TEM examinations of both species (S3 Fig), possibly resulting from the difference in density of abutment between the gelatinous material of adjacent cells. Thus, *V*. *reticuliferus* can be clearly distinguished from *V*. *africanus* by its reticulate zygote wall, and confluent or indistinct individual sheaths of spheroids. In addition, the presence of a single CBC in the most highly conserved region of nuclear rDNA ITS-2 secondary structure [39] between lineage RT (composed of three OTUs of *V*. *reticuliferus*, see below) and lineage AF (including *V*. *africanus* strain 2013-0703-VO4) (Fig 4) and lack of production of mature viable hybrid zygotes (representing hybrid inviability) in our intercross between the two species (S4 Fig) suggest sexual isolation between them. Thus, *V*. *reticuliferus* and *V*. *africanus* are morphologically and genetically different species.

Although zygote morphology is unknown in two heterothallic strains of “*V*. *africanus*” [UTEX 1890 and 1891 (Darra 4 and 6, respectively [14])] and “*V*. *africanus*” strain UTEX 2907, the three strains have indistinct individual sheaths of gelatinous matrix in the asexual spheroids as in *V*. *reticuliferus* strains 2013-0703-VO1, VO2 and VO3 (S1 and S2 Figs). In addition, these three UTEX strains and *V*. *reticuliferus* strains 2013-0703-VO1, VO2 and VO3 form a small clade (lineage RT, Fig 3) in which no CBC is detected in the most highly conserved region of the secondary structure of ITS-2 of nuclear rDNA (Fig 4; S5 Fig). Therefore, they can be identified as *V*. *reticuliferus*.

Starr [14] reported four types of sexual reproduction in “*V*. *africanus*” (S2 Table). Based on our study, the strains of heterothallic, dioecious type [Darra4 (UTEX 1890) and Darra6 (UTEX 1891)] were assigned to *V*. *reticuliferus*. However, strains of the other three sexual types exhibiting homothallic sexuality studied by Starr [14] are not available, but the nuclear rDNA ITS-2 phylogeny demonstrated that *V*. *africanus* strain 2013-0703-VO4 (homothallic) and strains of the other three sexual types (exhibiting homothallic sexuality) studied by Starr [14] form a clade that is sister to the clade of *V*. *reticuliferus* (lineage RT) (Fig 3). Within the homothallic clade, dioecious, homothallic type (UTEX 1889) is sister to the lineage AF (composed of the other three homothallic strains), and these two sister lineages show three CBCs in the most conserved region [39] of secondary structure of nuclear rDNA ITS-2 (Fig 4). Thus, this homothallic clade may be subdivided into at least two cryptic species when considering the ITS-2 sequence and types of sexual spheroids (Figs 3 and 4). However, other phenotypic data including zygote wall and gelatinous matrix morphology as well as other sequence data are now lacking, but are needed to taxonomically identify strain UTEX 1889 and two strains of lineage AF (strains UTEX 1893 and UTEX 1892).

West [41] reported “female” spheroids containing mature zygotes in the African material of *V*. *africanus*, but he did not observe male spheroids. Thus, these female spheroids may have actually been monoecious. According to Shaw [43], the Philippine *V*. *africanus* material produces both male and monoecious spheroids in the same parental spheroid as in our new strains and monoecious with males type described by Starr [14] (S1 Table). The Indian material of *V*. *africanus* examined by Iyengar [43] produces both dioecious (male and female) and monoecious sexual spheroids. Therefore, *V*. *africanus* may be a worldwide species with homothallic sexuality and smooth-walled zygotes. Further morphological and molecular studies using living strains of *V*. *africanus*-like species are needed to understand the evolution of homothallism and/or monoecism within this group and to more clearly delineate *V*. *reticuliferus* and *V*. *africanus*.

As in previous multigene phylogenies [5, 23, 24], our phylogenetic analyses robustly resolved that three volvocacean genera *Volvox*, *Pleodorina*, and *Eudorina* are not monophyletic, and the lectotype species *V*. *globator* [45] is robustly separated from *Volvox* sect. *Merrillosphaera* sensu Smith (Fig 2). Therefore, *V*. *africanus* and *V*. *reticuliferus* should not be classified to the genus *Volvox* when the generic classification is strictly based on the monophyletic concept. As discussed previously [5], however, division of the genus *Volvox* into four monophyletic genera (Fig 2) would not resolve problems for nonmonophyly of the genera *Eudorina* and *Pleodorina* [5, 24]. New phenotypic characters are still needed to clearly delineate monophyletic genera proposed in future within the Volvocaceae [5, 24]. Thus, division of the genus *Volvox* was not proposed here as suggested previously [5].

The present study delineates a new species of *Volvox* (*V*. *reticuliferus*) that could be assigned to the section *Merrillosphaera* sensu Smith [2]. However, this section is non-monophyletic as shown in Fig 2. Thus, in order to avoid discrepancy between phylogeny and section level classification of *Volvox* in future studies, here we propose a new classification system (four monophyletic sections) of *Volvox* at section level (S3 Table). Thus, *V*. *powersii* and *V*. *gigas* should be removed from section *Merrillosphaera* and assigned to section *Besseyosphaera* (type species: *V*. *powersii*), and section *Copelandosphaera* (type species: *V*. *dissipatrix*) should be synonymized under section *Merrillosphaera* based on the phylogenetic results (for details, see S3 Table and S1 File).

## Taxonomic Treatments

*Volvox* sect. *Merrillosphaera* (Shaw) Printz 1927: 59 [46].

Basionym: *Merrillosphaera* Shaw 1922: 118 [47].
Type species *V*. *carteri* Stein.
Heterotypic synonyms: *Volvox* sect. *Copelandosphaera* (Shaw) Printz 1927: 59 [46]; *Volvox* sect *Campbellosphaera* (Shaw) Printz 1927: 59 [46]; *Campbellosphaera* Shaw 1919: 510 [48]; *Copelandosphaera* Shaw 1922: 223 [47].

*Volvox reticuliferus* Nozaki sp. nov.

Asexual spheroids ovoid or ellipsoidal with broad anterior face, composed of 800–3000 cells, measuring up to 400 μm in length, with 2–4 gonidia distributed in the middle portion with or without an additional 1–4 gonidia in the posterior portion. Somatic cells nearly spherical in shape, lacking cytoplasmic bridges, embedded within individual sheaths of gelatinous or extracellular matrix, measuring up to 8 μm in diameter. Individual sheaths confluent or indistinct. Each somatic cell enclosed by a broad secondary boundary layer of gelatinous matrix. Gonidia vacuolated, with a large chloroplast, measuring up to 60 μm in diameter. The surface of the chloroplast of gonidia with fine striations radially arranged. Juvenile spheroids developing in pairs within the parent. Gonidia of the next generation evident during cell divisions of formation of juvenile spheroids. Sexual spheroids dioecious with production of male or female spheroids in a single genetic strain. Male spheroids ellipsoidal or cylindrical, containing 1000–1500 biflagellate somatic cells and 80–120 androgonidia. Androgonidia dividing into plate-shaped sperm packets. Female spheroids with 1000–2000 biflagellate somatic cells and 8–20 eggs. After possible fertilization, the zygote developing a reticulate cell wall and turning reddish brown in color. The mature zygote measuring 33–39 μm in diameter.

Holotype: Resin-embedded asexual spheroids of *Volvox reticuliferus* strain 2013-0703-VO2 (TNS-AL-58912), deposited in TNS (Department of Botany, National Museum of Nature and Science). This strain is available as NIES-3782 from the Microbial Culture Collection at National Institute for Environmental Studies (Kasai et al. 2009).
Strains examined: 2013-0703-VO1, 2013-0703-VO2, 2013-0703-VO3, VO123-F1-6, VO123-F1-7, UTEX 1890, UTEX 1891 and UTEX 2907 (S1 Table).
Etymology: The species epithet “*reticuliferus*” meaning having reticulations.
Type locality: Lake Biwa, Shiga Prefecture, Japan. A water sample was collected by FT and HN on 3 July 2013.
Distribution: Japan (the present study) and Australia [14].

## Key to the Sections Emended in the Genus *Volvox*

(1) Somatic cells in adult spheroids stellate or amoeboid in surface view and with thick cytoplasmic bridges between them; zygote walls spiny------- *V*. sect. *Volvox*
(1) Somatic cells in adult spheroids almost circular in surface view and lacking thick cytoplasmic bridges between them; zygote walls not spiny---------------------- (2)
(2) Asexual spheroid with more than 20 gonidia-------------------------------------------------------------------------------- *V*. sect. *Besseyosphaera* (Shaw) Printz
(2) Asexual spheroid with less than 20 gonidia----------------------------------- (3)
(3) Asexual spheroid with gelatinous mass radiating thin strands in the center---------------------------------------------------------- *V*. sect. *Janetosphaera* (Shaw) Printz
(3) Asexual spheroid without gelatinous mass radiating thin strands in the center------------------------------------------------------- *V*. sect. *Merrillosphaera* (Shaw) Printz

## Key to the Species of *Volvox* sect. *Merrillosphaera* Emended (Mainly Based on the Previous Key [5])

(1) Adult spheroids with cytoplasmic bridges between cells----------------------------------------------------------------------------------------- *V*. *dissipatrix* (Shaw) Printz
(1) Adult spheroids lacking cytoplasmic bridges between cells------------------------(2)
(2) Gonidia of the next generation evident during embryogenesis--------------(3)
(2) Gonidia of the next generation not evident during embryogenesis---------(6)
(3) Juvenile spheroids developing in pairs within the parent----------------------------(4)
(3) Juvenile spheroids not developing in pairs within the parent-----------------------(5)
(4) Individual sheaths distinct; zygote wall smooth------ *V*. *africanus* G. S. West
(4) Individual sheaths confluent or indistinct; zygote wall reticulate------------------------------------------------------------------- *V*. *reticuliferus* Nozaki sp. nov.
(5) Gonidia in posterior tier of asexual spheroid showing earlier development of juvenile spheroids than those of the rest-------------------- *V*. *obversus* (Shaw) Printz
(5) Gonidia in posterior tier of asexual spheroid not showing earlier development of juvenile spheroids than those of the rest------------------------------ *V*. *carteri* Stein
(6) Mature gonidia in asexual spheroid less than 50 μm in diameter-----------(7)
(6) Mature gonidia in asexual spheroid 50–55 μm in diameter--------------------------------------------------------*V*. *ovalis* Pocock ex Nozaki et A. W. Coleman
(7) Male spheroids composed of only androgonidia (sperm packets) ------------------------------------------------------------------------------------ *V*. *spermatosphaera* Powers
(7) Male spheroids composed of somatic cells and androgonidia (sperm packets) ------------------------------------------------------------------------------------ *V*. *tertius* Meyer

## Supporting Information

S1 Fig Nomarski interference microscopy of *Volvox africanus* G. S. West. (A) Surface view of asexual spheroids showing small somatic cells and larger reproductive cells (gonidia). (B) Somatic cells of asexual spheroid showing lack of cytoplasmic bridges between them. (C, D) Asexual spheroids stained with dilute aniline blue, showing angular individual sheaths compactly arranged. Note a broad secondary boundary layer (asterisk) surrounding each somatic cell within individual sheath. (E) Plakeal stage. Note morphological differentiation of gonidium initials of the next generation. (F) Development of asexual and male and monoecious spheroids in a single parental spheroid. (G) Mature male spheroid with sperm packets. (H, I) Monoecious spheroids with eggs and sperm packets. (J, K) Two views of mature zygotes with a smooth wall. Abbreviations: as, asexual spheroid; e, egg; g, gonidium or gonidium initial; is, individual sheath; ma, male sexual spheroid; mo, monoecious sexual spheroid; p, pyrenoid; sp, sperm packet. (B-E, H) Strain 2013-0703-VO4. (A, F, G, I-K) Strain VO4-F1-1.
(TIF)

S2 Fig Light microscopy of asexual spheroids of three UTEX strains of “*Volvox africanus*.” (A) UTEX 1890 (= Darra 4, Starr 1971). (B) UTEX 1891 (= NIES-863, Darra 6, Starr 1971). (C) UTEX 2907. Stained with dilute aniline blue, showing a broad secondary layer (asterisk) of the gelatinous matrix surrounding each somatic cell. All at the same magnification. Note that angular individual sheaths are indistinct.
(TIF)

S3 Fig Transmission electron micrographs of asexual spheroids of *Volvox reticuliferus* Nozaki sp. nov. and *V*. *africanus* G. S. West. (A–C) *V*. *reticuliferus* strain 2013-0703-VO2. Asterisks indicate broad secondary boundary layer of the extracellular matrix. (D–F) *V*. *africanus* strain 2013-0703-VO4. Note that individual sheaths are evident. Abbreviations: c, chloroplast; e, cellular envelope of the extracellular matrix enclosing protoplast tightly; is, individual sheath; m, mitochondrion; n, nucleus; p, pyrenoid. Arrows indicate tripartite colonial boundary of the extracellular matrix encompassing the whole spheroids.
(TIF)

S4 Fig Results of intercrossing between *Volvox reticuliferus* Nozaki sp. nov. and *V*. *africanus* G. S. West. (A, D) Seven days after the intercrossing. (B, E) Eleven days after the intercrossing. (C, F) Twenty-one days after the intercrossing. Note walled eggs or possible hybrid zygotes formed within female spheroids of *V*. *reticuliferus* strain VO123-F1-6 after being mixed with isolated male spheroids of *V*. *africanus* strain VO4-F1-1.
(TIF)

S5 Fig Secondary structures of nuclear rDNA ITS-2 transcripts *Volvox reticuliferus* Nozaki sp. nov., *V*. *africanus* G. S. West and *V*. *africanus*-like UTEX strains. (A) *V*. *reticuliferus* strain 2013-0703-VO2. Nuclear rDNA ITS-2 sequences of the three strains of *V*. *reticuliferus* (2013-0703-VO1, VO2 and VO3) are identical. Differences among strains within the lineage RT (Fig 3) [vs. “*V*. *africanus*” strains UTEX 1890, UTEX 1891 (= NIES-863), and UTEX 2907] are indicated by characters just outside the secondary structure. Single asterisk means that the difference was detected only in strain UTEX 2907, and double asterisks imply that the difference was recognized only in strains UTEX 1890 and UTEX 1891, of which nuclear rDNA ITS-2 sequences are identical. (B) *V*. *africanus* strain 2013-0703-VO4. Note the U-U mismatch in helix II (arrowheads) and the modified YGGY motif (GGU or UGGGU) on the 5’ side of helix III (boldface). Differences among the strains belonging to lineage AF (Fig 3) are shown by characters just outside the secondary structure. Single asterisk means that the difference was detected only in “*V*. *africanus*” strain UTEX 1893, and double asterisks imply that the difference was detected only in “*V*. *africanus*” strain UTEX 1892. (C) “*V*. *africanus*” strain UTEX 1889.
(PDF)

S1 File Remarks of four sections of the genus *Volvox* proposed here. (PDF)

S1 Table List of *Volvox* species/strains included in the phylogenetic analyses of ITS-2 sequences (Fig 3) and DDBJ/EMBL/GENBANK accession numbers. (DOCX)

S2 Table Comparison of various materials of *Volvox africanus* G. S. West and *V*. *reticuliferus* Nozaki sp. nov. (DOCX)

S3 Table Revised taxonomic system of sections of the genus *Volvox*. (DOCX)
