# Supplementary figures and images for: Delineating a New Heterothallic Species of Volvox (Volvocaceae, Chlorophyceae) Using New Strains of “Volvox africanus”
Source: PLoS One. 2015 Nov 12;10(11):e0142632. doi: 10.1371/journal.pone.0142632 (PMC4643018; doi:10.1371/journal.pone.0142632)

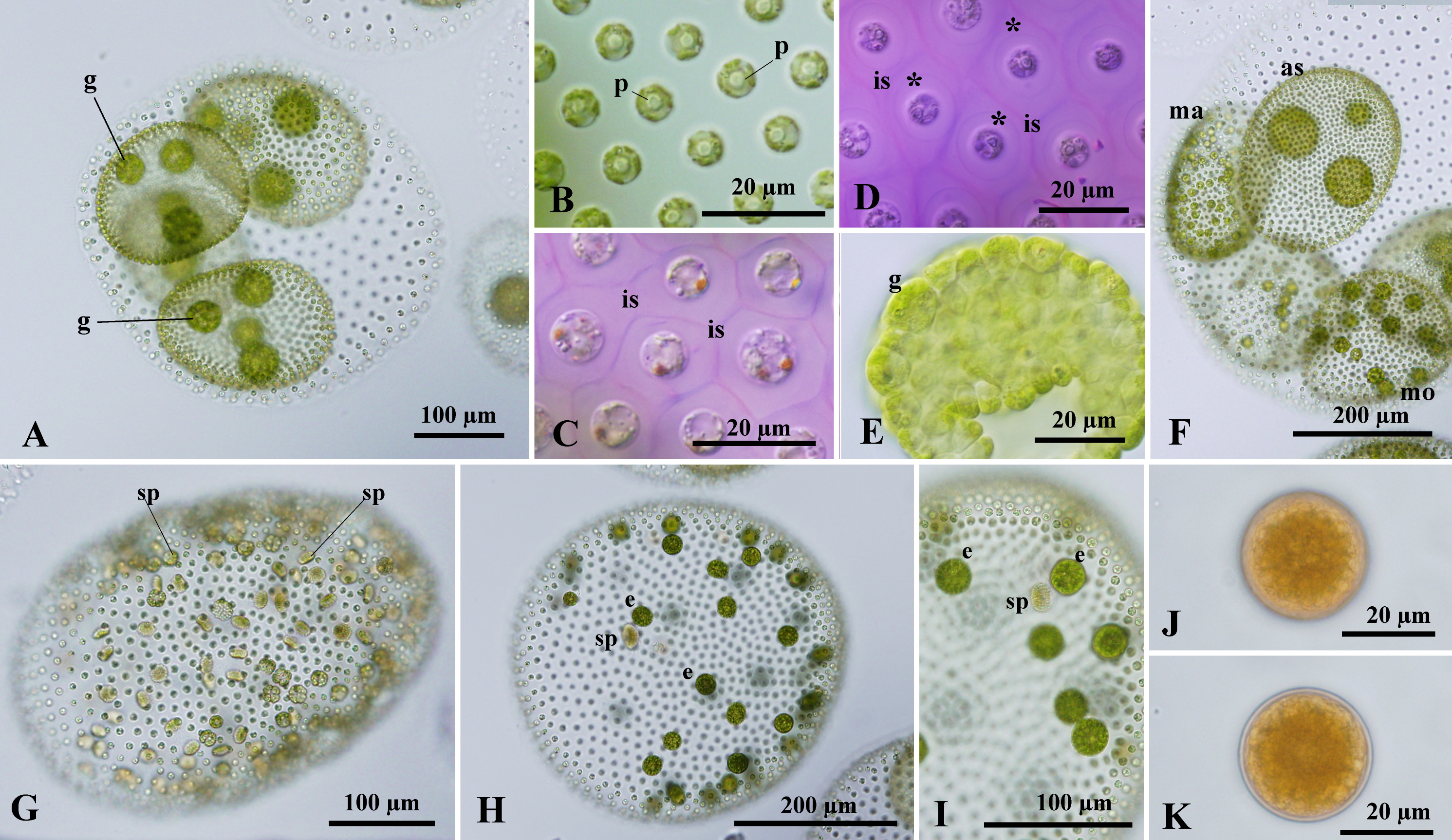

Supplement: S1 Fig — (A) Surface view of asexual spheroids showing small somatic cells and larger reproductive cells (gonidia). (B) Somatic cells of asexual spheroid showing lack of cytoplasmic bridges between them. (C, D) Asexual spheroids stained with dilute aniline blue, showing angular individual sheaths compactly arranged. Note a broad secondary boundary layer (asterisk) surrounding each somatic cell within individual sheath. (E) Plakeal stage. Note morphological differentiation of gonidium initials of the next generation. (F) Development of asexual and male and monoecious spheroids in a single parental spheroid. (G) Mature male spheroid with sperm packets. (H, I) Monoecious spheroids with eggs and sperm packets. (J, K) Two views of mature zygotes with a smooth wall. Abbreviations: as, asexual spheroid; e, egg; g, gonidium or gonidium initial; is, individual sheath; ma, male sexual spheroid; mo, monoecious sexual spheroid; p, pyrenoid; sp, sperm packet. (B-E, H) Strain 2013-0703-VO4. (A, F, G, I-K) Strain VO4-F1-1. (TIF) [file pone.0142632.s001.tif]

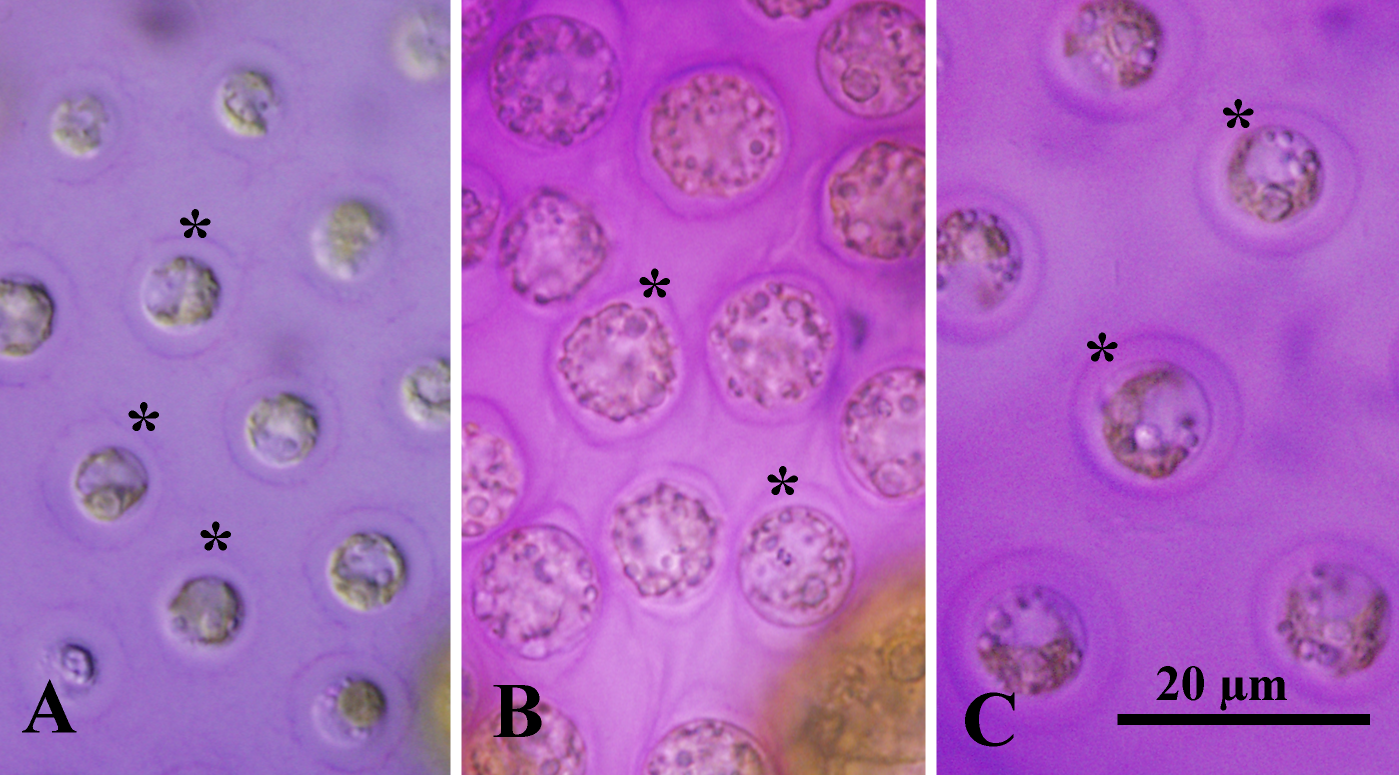

Supplement: S2 Fig — (A) UTEX 1890 (= Darra 4, Starr 1971). (B) UTEX 1891 (= NIES-863, Darra 6, Starr 1971). (C) UTEX 2907. Stained with dilute aniline blue, showing a broad secondary layer (asterisk) of the gelatinous matrix surrounding each somatic cell. All at the same magnification. Note that angular individual sheaths are indistinct. (TIF) [file pone.0142632.s002.tif]

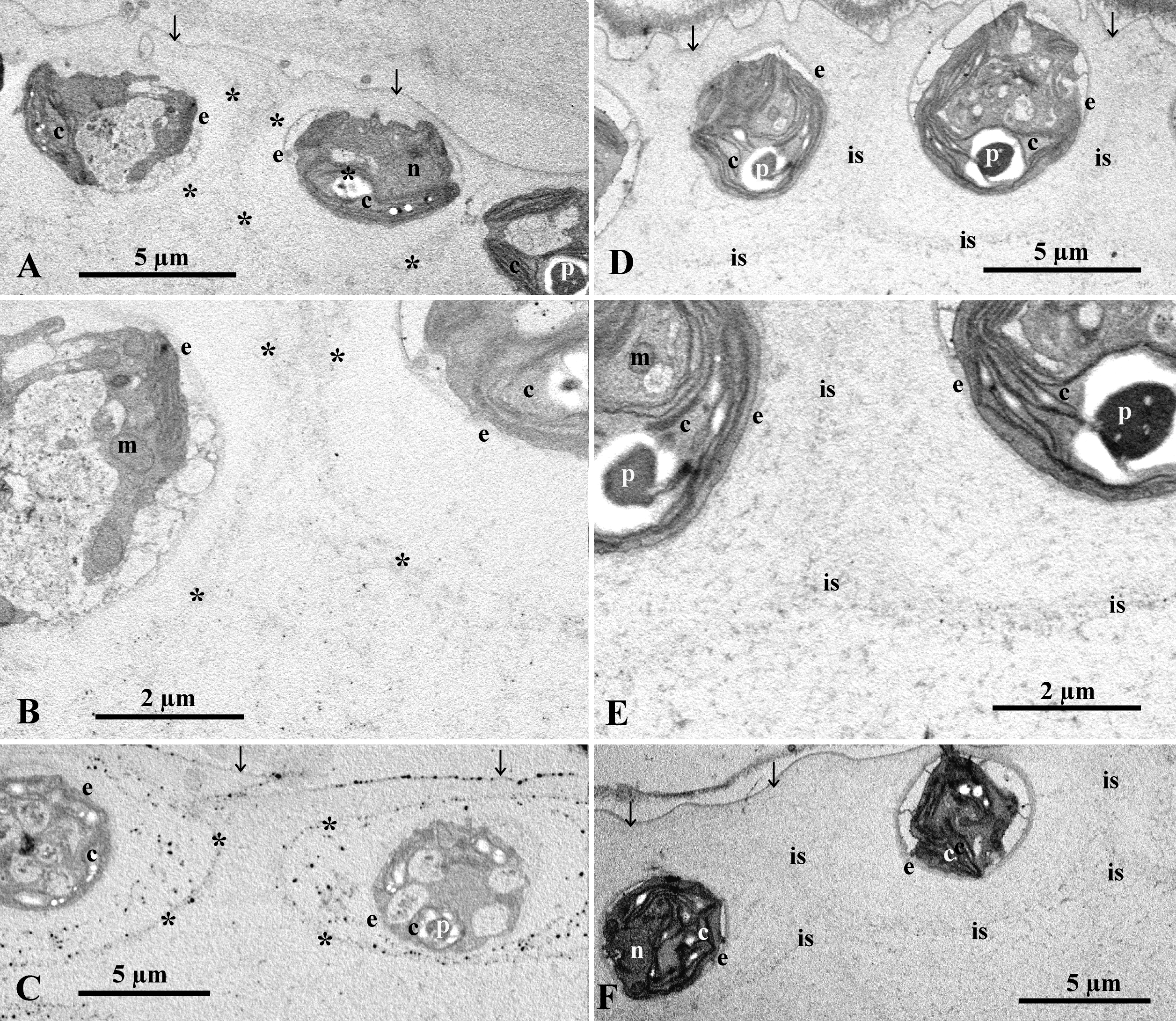

Supplement: S3 Fig — (A–C) V. reticuliferus strain 2013-0703-VO2. Asterisks indicate broad secondary boundary layer of the extracellular matrix. (D–F) V. africanus strain 2013-0703-VO4. Note that individual sheaths are evident. Abbreviations: c, chloroplast; e, cellular envelope of the extracellular matrix enclosing protoplast tightly; is, individual sheath; m, mitochondrion; n, nucleus; p, pyrenoid. Arrows indicate tripartite colonial boundary of the extracellular matrix encompassing the whole spheroids. (TIF) [file pone.0142632.s003.tif]

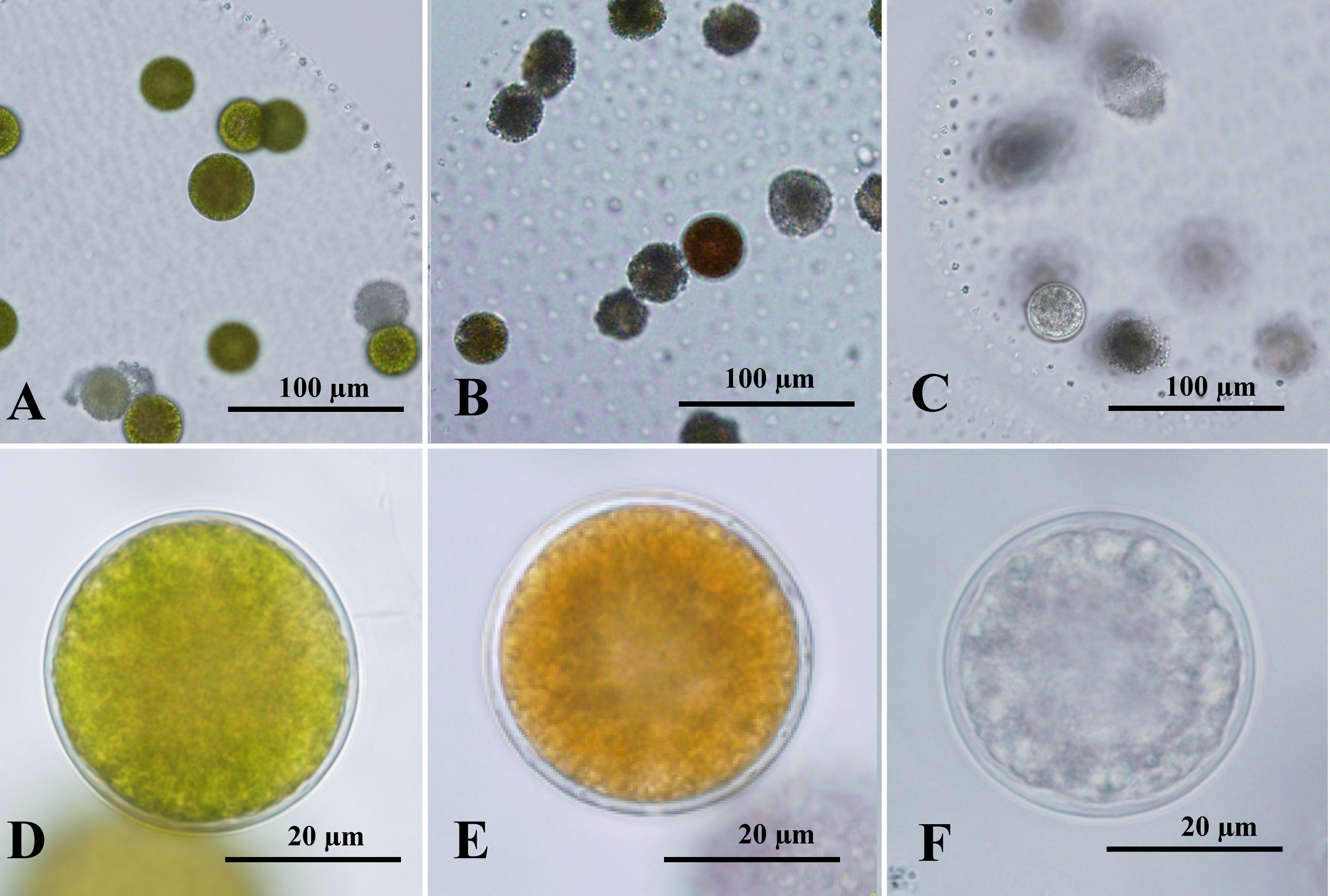

Supplement: S4 Fig — (A, D) Seven days after the intercrossing. (B, E) Eleven days after the intercrossing. (C, F) Twenty-one days after the intercrossing. Note walled eggs or possible hybrid zygotes formed within female spheroids of V. reticuliferus strain VO123-F1-6 after being mixed with isolated male spheroids of V. africanus strain VO4-F1-1. (TIF) [file pone.0142632.s004.tif]

**A**

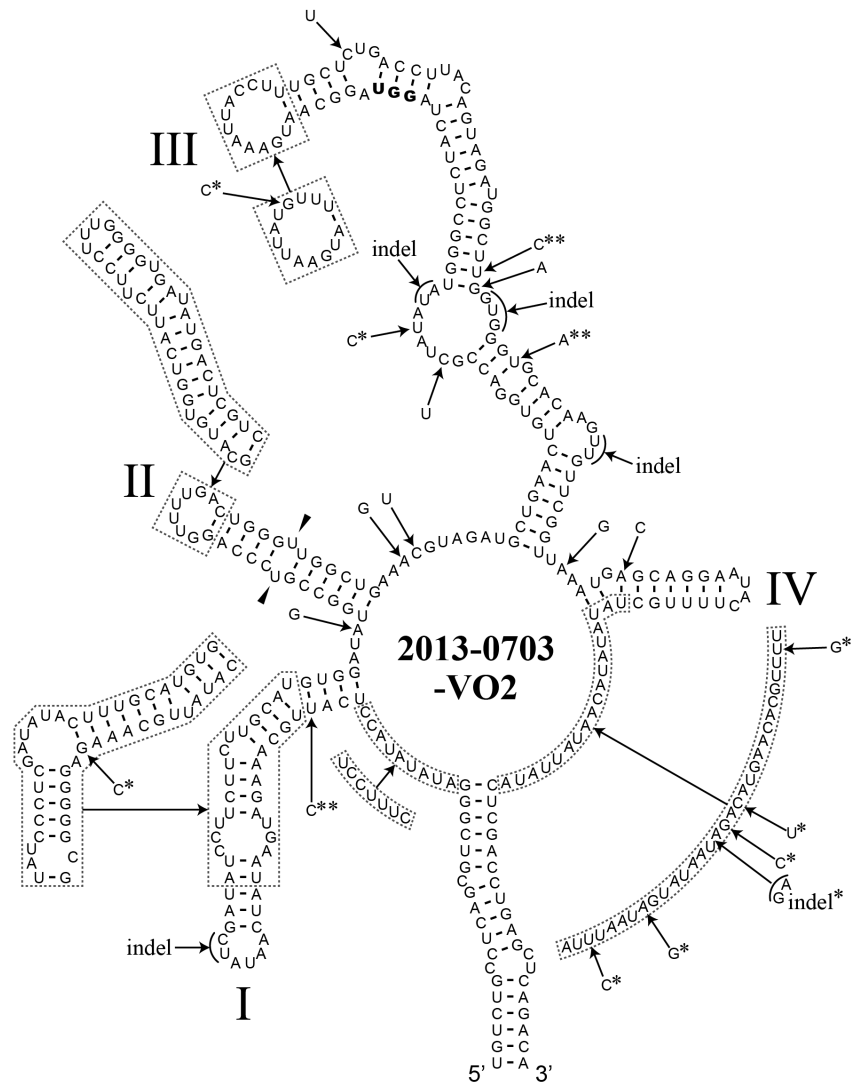

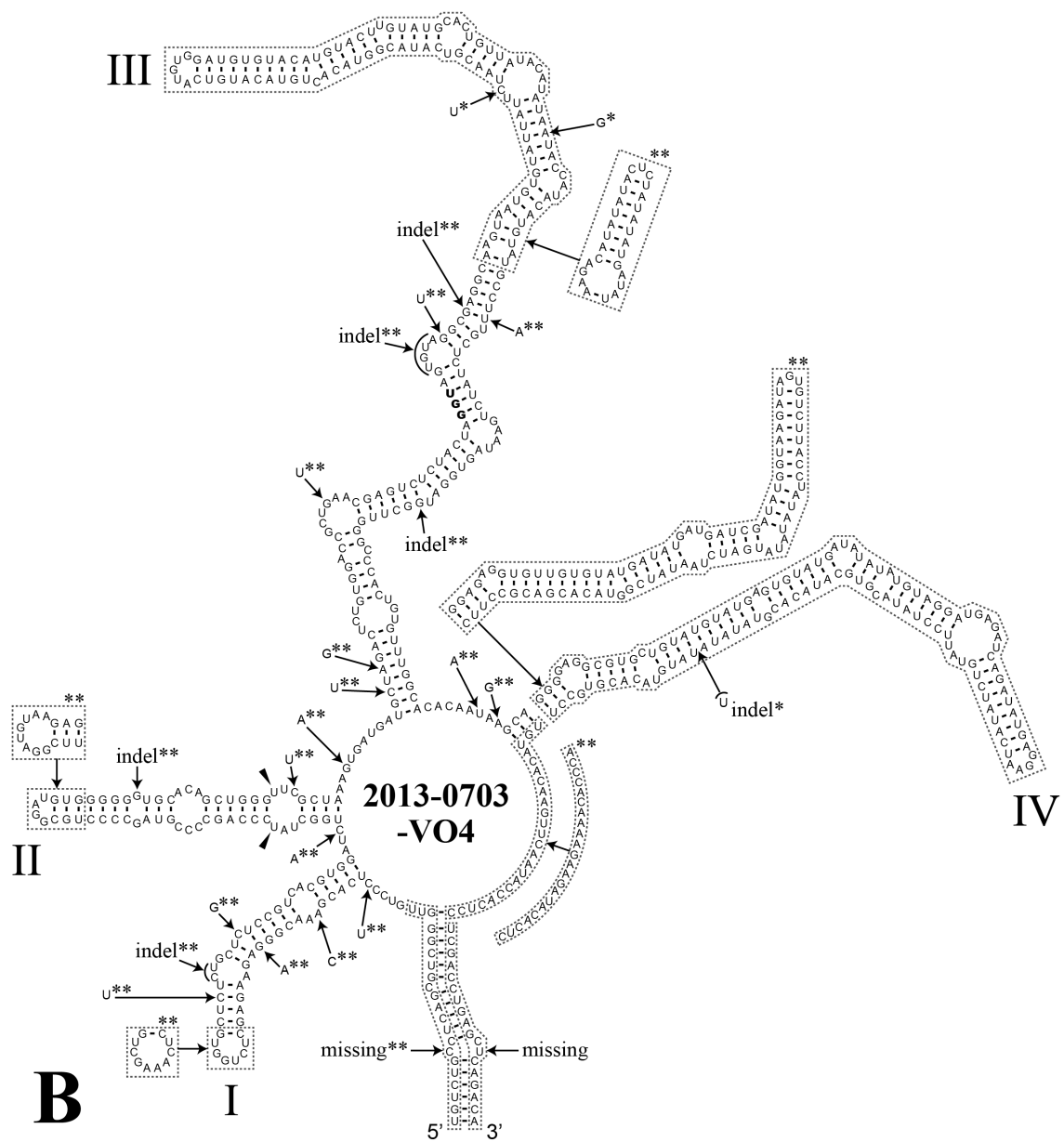

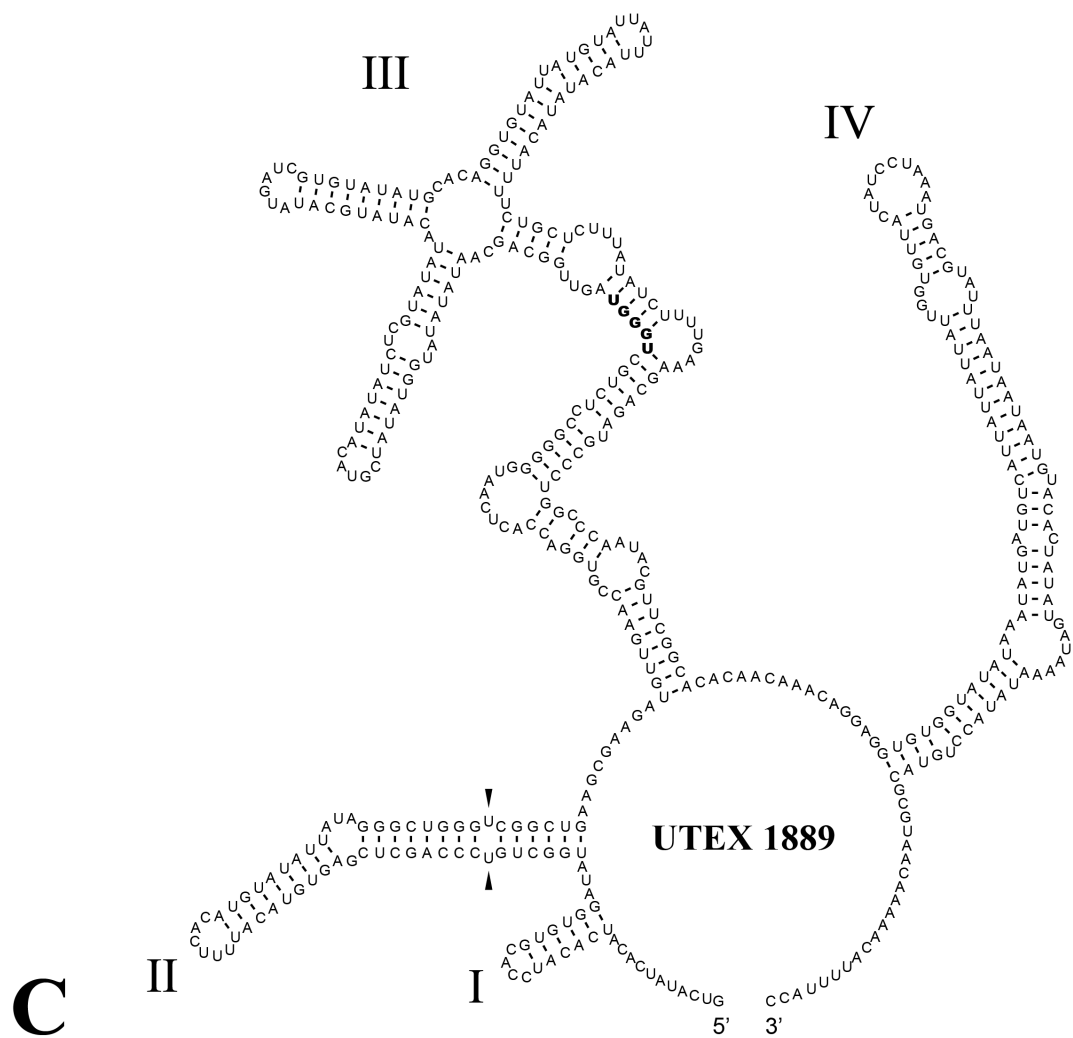

Supplement: S5 Fig — (A) V. reticuliferus strain 2013-0703-VO2. Nuclear rDNA ITS-2 sequences of the three strains of V. reticuliferus (2013-0703-VO1, VO2 and VO3) are identical. Differences among strains within the lineage RT (Fig 3) [vs. “V. africanus” strains UTEX 1890, UTEX 1891 (= NIES-863), and UTEX 2907] are indicated by characters just outside the secondary structure. Single asterisk means that the difference was detected only in strain UTEX 2907, and double asterisks imply that the difference was recognized only in strains UTEX 1890 and UTEX 1891, of which nuclear rDNA ITS-2 sequences are identical. (B) V. africanus strain 2013-0703-VO4. Note the U-U mismatch in helix II (arrowheads) and the modified YGGY motif (GGU or UGGGU) on the 5’ side of helix III (boldface). Differences among the strains belonging to lineage AF (Fig 3) are shown by characters just outside the secondary structure. Single asterisk means that the difference was detected only in “V. africanus” strain UTEX 1893, and double asterisks imply that the difference was detected only in “V. africanus” strain UTEX 1892. (C) “V. africanus” strain UTEX 1889. (PDF) [file pone.0142632.s005.pdf]
