# Supplementary material for: Delineating a New Heterothallic Species of Volvox (Volvocaceae, Chlorophyceae) Using New Strains of “Volvox africanus”
Source: PLoS One. 2015 Nov 12;10(11):e0142632. doi: 10.1371/journal.pone.0142632 (PMC4643018; doi:10.1371/journal.pone.0142632)
Supplement: S1 File — (PDF) [file pone.0142632.s006.pdf]

S1 File. Remarks of four sections of the genus *Volvox* proposed here.

Section *Volvox* (lectotype species: *V. globator* Linnaeus)

This group corresponds to section "*Euvolvox*" by Smith [1] and is characterized by having thick cytoplasmic bridges between cells in adult spheroids and spiny cell walls in the zygotes. These two characters are very distinct within the colonial Volvocales [1-3]. Phylogenetic analyses resolve robust monophyly of this section [3, 4] (Fig. 2).

Section *Merrillosphaera* (Shaw) Printz (type species: *V. carteri* Stein)

Synonyms: Section *Copelandosphaera* (Shaw) Printz (type species: *V. dissipatrix* (Shaw) Printz)

Section *Campbellosphaera* (Shaw) Printz (type species: *V. obversus* (Shaw) Printz)

Based on the molecular phylogeny, *V. dissipatrix* (the type species of section *Copelandosphaera*, one of the four sections of Smith [1]) forms a large clade with species of section *Merrillosphaera* sensu Smith (1944) excluding *V. gigas* Pocock and *V. powersii* (Shaw) Printz. The latter two species are distinctive in having more than 20 gonidia in an asexual spheroid [1, 5]. *V. dissipatrix* has thin cytoplasmic bridges between cells in adult spheroids as in *V. aureus* Ehrenberg [1]. However, the spheroid of *V. aureus* has a gelatinous core radiating thin strands in the center, but such a structure is not present in other sections of *Volvox* [1, 5-7]. Thus, section *Merrillosphaera* should be characterized by having an asexual spheroid with less than 20 gonidia, lacking thick cytoplasmic bridges between adult cells in the spheroid and lacking a gelatinous core with radiating thin strands in the center of the spheroid. Although Smith [1] characterized section *Merrillosphaera* and *Janetosphaera* by having distinct and confluent (indistinct) individual sheaths, respectively, of the peripheral region of the gelatinous matrix of spheroids, the present study demonstrated that these two types of individual sheaths are recognized in the two closely related species, *V. africanus* G. S. West and *V. reticuliferus* Nozaki sp. nov., within section *Merrillosphaera* emended (Figs. 1 and 2; S1 Fig.).

Section *Besseyosphaera* (Shaw) Printz (type species: *V. powersii*)

Smith (1944) assigned *V. powersii* and *V. gigas* to section *Merrillosphaera* because these two species lack cytoplasmic bridges between cells in an adult spheroids. However, molecular phylogenetic analyses demonstrated that these two species form a small monophyletic group or one of the four separate clades constituting the genus *Volvox* [5, 8] (Fig. 2). These two species are unique among *Volvox* in having more than 20 gonidia in an asexual spheroid [1]). Thus, these two species are now assigned to section *Besseyosphaera* (Shaw) Printz [9].

Section *Janetosphaera* (Shaw) Printz (type species *V. aureus*)

Although Starr [10] described *V. pocockiae* Starr in section *Janetosphaera*, based on the fact that the spheroid has thin cytoplasmic bridges between adult cells and the gelatinous or extracellular matrix structure of the spheroid is similar to that of *V. aureus*. However, the phylogeny of ITS-2 of nuclear rDNA does not show *V. pocockiae*'s related species [11], and the culture strain of *V. pocockiae* (UTEX 1872 [12]) is not now available.

References

1. Smith GM. A comparative study of the species of *Volvox*. Trans. Am. Microsc. Soc. 1944; 63: 265-310.
2. Nozaki H, Ito M Phylogenetic relationships within the colonial Volvocales (Chlorophyta) inferred from cladistic analysis based on morphological data. J. Phycol. 1994; 30: 353-365.
3. Nozaki H, Yamada TK, Takahashi F, Matsuzaki R, Nakada T. New "missing link" genus of the colonial volvocine green algae gives insights into the evolution of oogamy. BMC Evol. Biol. 2014; 14: 37.
4. Nozaki H, Misawa K, Kajita T, Kato M, Nohara S, Watanabe MM. Origin and evolution of the colonial Volvocales (Chlorophyceae) as inferred from multiple, chloroplast gene sequences. Mol. Phylog. Evol. 2000; 17: 256-268.
5. Nozaki H, Coleman AW. A new species of *Volvox* sect. *Merrillosphaera* (Volvocaceae, Chlorophyceae) from Texas. J. Phycol. 2011;47: 673-679.
6. Isaka N, Kawai-Toyooka H, Matsuzaki R, Nakada T, Nozaki H. Description of two new monoecious species of *Volvox* sect. *Volvox* (Volvocaceae, Chlorophyceae), based on comparative morphology and molecular phylogeny of cultured material. J. Phycol. 2012; 48:759-767.
7. Nozaki H, Ueki N, Misumi O, Yamamoto K, Yamashita S, Herron MD et al. Morphology and reproduction of *Volvox capensis* (Volvocales, Chlorophyceae) from Montana, USA. Phycologia 2015; 54: 316-320.
8. Nozaki H. Origin and evolution of the genera *Pleodorina* and *Volvox* (Volvocales). Biologia 2003; 58: 425-431.
9. Printz H. Chlorophyceae (nebst Conjugatae, Heterokontae und Charophyta). Vol. 2, Auf. 3. In Engler A, Prantl K, editors. Die natürlichen Pflanzenfamilien. Leipzig: Wilhelm Engelmann; 1927. pp. 1-463.
10. Starr RC. *Volvox pocockiae*, a new species with dwarf males. J. Phycol. 1970; 6: 234-239.
11. Coleman AW. Phylogenetic analysis of "Volvocacae" for comparative genetic studies. Proc. Natl. Acad. Sci. USA 1999; 96: 13892-13897.
12. Starr RC, Zeikus JA. UTEX - The Culture Collection of Algae at the University of Texas at Austin. J. Phycol. 1993; 29 (2), Supplement: 1-106.
