# Supplementary material for: Delineating a New Heterothallic Species of Volvox (Volvocaceae, Chlorophyceae) Using New Strains of “Volvox africanus”
Source: PLoS One. 2015 Nov 12;10(11):e0142632. doi: 10.1371/journal.pone.0142632 (PMC4643018; doi:10.1371/journal.pone.0142632)
Supplement: S1 Table — (DOCX) [file pone.0142632.s007.docx]

## S1 Table. List of *Volvox* species/strains included in the phylogenetic analyses of ITS-2 sequences (with DDBJ/EMBL/GENBANK accession numbers; Fig. 3) and/or morphological observations (Fig. 1; S1 Fig.).

| Species | Strain designation | Origin | Sexual type^1^ | Accession no. | Reference |
| --- | --- | --- | --- | --- | --- |
| *V. africanus* | 2013-0703-VO4  (=NIES-3780) | Lake Biwa, Japan | Monoecious with males, homothallic | LC090159^2^ | The present study |
| *V. africanus* | VO4-F1-1  (=NIES-3784) | F_1_ progeny strain of 2013-0703-VO4 | Monoecious with males, homothallic |  | The present study |
| “*V. africanus*” | Mo-1-Ea  (= UTEX^3^ 1889) | A shallow pond near Rocheport, MO, USA | Dioecious, homothallic | U67008 | [1, 2] |
| “*V. africanus*” | Ecca Pass 3  (=UTEX 1892) | Ecca Pass near Grahamstown, South Africa | Monoecious,homothallic | U67011 | [1, 2] |
| “*V. africanus*” | India 65-26  (=UTEX 1893) | A dry pond at Nadi near Santipura, West Bengal, India | Monoecious with males, homothallic | AF182440 | [1, 2] |
| *V. reticuliferus* | 2013-0703-VO1  (=NIES-3781) | Lake Biwa, Japan | Dioecious, heterothallic, male | LC090160^2^ | The present study |
| *V. reticuliferus* | 2013-0703-VO2  (=NIES-3782) | Lake Biwa, Japan | Dioecious, heterothallic, female | LC090161^2^ | The present study |
| *V. reticuliferus* | 2013-0703-VO3  (=NIES-3783) | Lake Biwa, Japan | Dioecious, heterothallic, male | LC090162^2^ | The present study |
| *V. reticuliferus* | VO123-F1-6  (=NIES-3785) | F_1_ progeny strain of 2013-0703-VO1 x VO2 x VO3 | Dioecious, heterothallic, female |  | The present study |
| *V. reticuliferus* | VO123-F1-7  (=NIES-3786) | F_1_ progeny strain of 2013-0703-VO1 x VO2 x VO3 | Dioecious, heterothallic, male |  | The present study |
| *V. reticuliferus* | “*V. africanus*” Darra 4 (= UTEX 1890) | A small pond in Darra, Queensland, Australia | Dioecious, heterothallic, male | AB771951 | [1, 3]  The present study |
| *V. reticuliferus* | “*V. africanus*”　Darra 6 (= UTEX 1891, NIES^4^-863) | A small pond in Darra, Queensland, Australia | Dioecious, heterothallic, female | AB771952 | [1, 3]  The present study |
| *V. reticuliferus* | “*V. africanus*”　UTEX 2907 | Unknown | Unknown | LC090163^2^ | The present study |
| *V. ovalis* | NIES-2569 |  |  | AB592343 |  |
| *V. tertius* | NIES-544 |  |  | AB592344 |  |
| *V. spermato-*  *sphaera* | UTEX 2273 |  |  | U67026 |  |
| *V. dissipatrix* | UTEX 1871 |  |  | U67020 |  |

^1^Based on Starr [1].

^2^Sequenced in the present study.

^3^Culture Collection of Algae at the University of Texas at Austin [4].

^4^Microbial Culture Collection at the National Institute for Environmental Studies [5].

**References**

1. Starr RC. Sexual reproduction in *Volvox africanus*. In: Parker BC, Brown Jr. RM, editors. Kansas: Contribution in Phycology, Allen Press; 1971. pp. 59-66.

2. Coleman AW. Phylogenetic analysis of "Volvocacae" for comparative genetic studies. Proc. Natl. Acad. Sci. USA 1999; 96: 13892-13897.

3. Hiraide R, Kawai-Toyooka H, Hamaji T, Matsuzaki R, Kawafune K, Abe J et al. The evolution of male-female sexual dimorphism predates the gender-based divergence of the mating locus gene *MAT3/RB*. Mol. Biol. Evol. 2013; 30: 1038-1040.

4. Starr RC, Zeikus JA. UTEX - The Culture Collection of Algae at the University of Texas at Austin. J. Phycol. 1993; 29 (2), Supplement: 1-106.

5. Kasai F, Kawachi M, Erata M, Mori F, Yumoto K, Sato M et al. editors. NIES-Collection. List of Strains. 8^th^ Edition. Jpn. J. Phycol. 2009; 57 (1), Supplement: 1-350, plates 1-7.
