# Supplementary material for: Delineating a New Heterothallic Species of Volvox (Volvocaceae, Chlorophyceae) Using New Strains of “Volvox africanus”
Source: PLoS One. 2015 Nov 12;10(11):e0142632. doi: 10.1371/journal.pone.0142632 (PMC4643018; doi:10.1371/journal.pone.0142632)
Supplement: S2 Table — (DOCX) [file pone.0142632.s008.docx]

**S2 Table.** Comparison of various materials of *Volvox* *africanus* G. S. West and *V.* *reticuliferus* Nozaki sp. nov.

| Species | *V. africanus* | *V. africanus* | *V. africanus* | *V. africanus* | *V. africanus* | “*V. africanus*” | “*V. africanus*” | “*V. africanus*” | *V. africanus* | *V. reticuliferus* | *V. reticuliferus* |
| --- | --- | --- | --- | --- | --- | --- | --- | --- | --- | --- | --- |
| Origin [culture strains] | Lake Albert Nyanza, on the border between Uganda and Congo, Africa | Small ponds in Ussangu Desert in the African region, formerly "German East Africa” | Fresh- water pools near Manila, Philip- pines | A small pool on the top of the Nandhi Hill, Mysore Province, India | A pool near the wagon road on  the Linyanti River, South Africa | A shallow pond near Rocheport, MO, USA [Mo-1-Eca (= UTEX 1889)] | Ecca Pass near Graham- stown, South Africa [Ecca Pass 3  (=UTEX 1892)] | A dry pond at Nadi near Santipura, West Bengal, India [India 65-26 (= UTEX 1893)] | Lake Biwa, Japan [2014-0703-VO4] | A small pond in Darra, Queensland,　Australia [Darra 4 (= UTEX 1890), Darra 6 (= UTEX 1891)] | Lake Biwa, Japan [2014-0703- VO1~3] |
| ITS2 of rDNA  supporting species identifi-  cation | Absent | Absent | Absent | Absent | Absent | Present | Present | Present | Present | Present | Present |
| Individual sheaths of asexual spheroid | ND | ND | Distinct | Distinct | Distinct | ND | ND | ND | Distinct | Confluent or indistinct | Confluent or indistinct |
| Sexual spheroids | A male spheroid of an unidentified *Volvox* species | Possible female spheroids containing mature zygotes | Both male and mono- ecious spheroids formed in the same parental spheroid | Dioecious (male and female) and mono- ecious spheroids | ND | Dioecious, male and female spheroids formed in the same strain  (homo-  thallic) | Mono- ecious spheroids | Both male and mono- ecious spheroids formed in the same parental spheroid | Both male and mono- ecious spheroids formed in the same parental spheroid | Dioecious, male and female spheroids formed in the different strains  (hetero-  thallic) | Dioecious, male and female spheroids formed in the different strains  (hetero-  thallic) |
| Zygote wall | ND | Smooth | Smooth | Smooth | ND | ND | ND | ND | Smooth | ND | Reticulate |
| Reference | [1] | [2] | [3, 4] | [4] | [5] | [6-8] | [6-8] | [6-8] | The present study | [6-9]  The present study | The present study |

**References**

1. West GS. Some new African species of *Volvox*. J. Quekett Microsc. Club (ser. 2) 1910; 11: 99-104.

2. West GS. A further contribution to our knowledge of the two African species of *Volvox*. J. Quekett Microsc. Club (ser. 2) 1918; 13: 425-438.

3. Shaw WR. *Merrillosphaera african*a at Manila. Philipp. J. Sci. 1923; 22: 185–218.

4. Iyengar MOP. Contribution to our knowledge of the colonial Volvocales of South India. J. Linn. Soc. Bot. 1933; 49: 323-373.

5. Rich F, Pocock MA. Observations on the genus *Volvox* in Africa. Ann. S. Afr. Mus. 1933; 16: 427-471, pls. 9-24.

6. Starr RC. Sexual reproduction in *Volvox africanus*. In: Parker BC, Brown Jr. RM, editors. Kansas: Contribution in Phycology, Allen Press; 1971. pp. 59-66.

7. Starr RC, Zeikus JA. UTEX - The Culture Collection of Algae at the University of Texas at Austin. J. Phycol. 1993; 29 (2), Supplement: 1-106.

8. Coleman AW. Phylogenetic analysis of "Volvocacae" for comparative genetic studies. Proc. Natl. Acad. Sci. USA 1999; 96: 13892-13897.

9. Hiraide R, Kawai-Toyooka H, Hamaji T, Matsuzaki R, Kawafune K, Abe J et al. The evolution of male-female sexual dimorphism predates the gender-based divergence of the mating locus gene *MAT3/RB*. Mol. Biol. Evol. 2013; 30: 1038-1040.
