# Supplementary material for: Delineating a New Heterothallic Species of Volvox (Volvocaceae, Chlorophyceae) Using New Strains of “Volvox africanus”
Source: PLoS One. 2015 Nov 12;10(11):e0142632. doi: 10.1371/journal.pone.0142632 (PMC4643018; doi:10.1371/journal.pone.0142632)
Supplement: S3 Table — (DOCX) [file pone.0142632.s009.docx]

**S3 Table.** Revised taxonomic system of sections of the genus *Volvox*.

| Section | *Volvox* | *Merrillosphaera* (Shaw) Printz | *Besseyosphaera*  (Shaw) Printz | *Janetosphaera* (Shaw) Printz |
| --- | --- | --- | --- | --- |
| Synonymous section |  | *Copelandosphaera* (Shaw) Printz  *Campbellosphaera* (Shaw) Printz |  |  |
| Species included based on morphological and molecular data | *V. globator* (type species of *Volvox*)  *V. capensis*  *V. barberi*  *V. rousseletii*  *V. kirkiorum*  *V. ferrisii* | *V. carteri* (type species of *Merrillosphaera*)  *V. obversus* (type species of *Campbellosphaera*)  *V. tertius*  *V. spermatosphaera*  *V. ovalis*  *V. africanus*  *V. reticuliferus*  *V. dissipatrix* (type species of *Copelandosphaera*) | *V. powersii* (type species of *Besseyosphaera*)  *V. gigas* | *V. aureus* (type species of *Janetosphaera*) |
| Species included based on only morphological data | *V. merrilli*  *V. perglobator*  *V. amboensis*  *V. prolificus* |  |  | *V. pocockiae* |
| Reference | [1-3] | [1, 4, 5]  The present study | [1, 6]  The present study | [1, 6, 7] |

**References**

1. Smith GM. A comparative study of the species of *Volvox*. Trans. Am. Microsc. Soc*.* 1944; 63: 265-310.

2. Isaka N, Kawai-Toyooka H, Matsuzaki R, Nakada T, Nozaki H. Description of two new monoecious species of *Volvox* sect. *Volvox* (Volvocaceae, Chlorophyceae), based on comparative morphology and molecular phylogeny of cultured material. J. Phycol. 2012; 48:759-767.

3. Nozaki H, Ueki N, Misumi O, Yamamoto K, Yamashita S, Herron MD et al. Morphology and reproduction of *Volvox capensis* (Volvocales, Chlorophyceae) from Montana, USA. Phycologia 2015; 54: 316-320.

4. Nozaki H. Morphology, sexual reproduction and taxonomy of *Volvox carteri* f. *kawasakiensis* f. nov. (Chlorophyta) from Japan. Phycologia 1988; 27: 209-220.

5. Nozaki H, Ueki N, Misumi O, Yamamoto K, Yamashita S, Herron MD et al. Morphology and reproduction of *Volvox capensis* (Volvocales, Chlorophyceae) from Montana, USA. Phycologia 2015; 54: 316-320.

6. Nozaki H. Origin and evolution of the genera *Pleodorina* and *Volvox* (Volvocales). Biologia 2003; 58: 425-431.

7. Starr RC. *Volvox pocockiae*, a new species with dwarf males. J. Phycol. 1970; 6: 234-239.
